# Alzheimer’s Disease and Diabetes Mellitus in Comparison: The Therapeutic Efficacy of the Vanadium Compound

**DOI:** 10.3390/ijms222111931

**Published:** 2021-11-03

**Authors:** Zhijun He, Guanying You, Qiong Liu, Nan Li

**Affiliations:** 1 College of Life Sciences and Oceanography, Shenzhen University, Shenzhen 518055, China; hezj@email.szu.edu.cn (Z.H.); 2060251029@email.szu.edu.cn (G.Y.); liuqiong@szu.edu.cn (Q.L.); 2 Shenzhen-Hong Kong Institute of Brain Science-Shenzhen Fundamental Research Institutions, Shenzhen 518055, China; 3 Shenzhen Bay Laboratory, Shenzhen 518055, China

**Keywords:** vanadium, bis(ethylmaltolato)oxidovanadium (IV) (BEOV), diabetes mellitus Alzheimer’s disease, peroxisome proliferator-activated receptor (PPARγ)

## Abstract

Alzheimer’s disease (AD) is an intractable neurodegenerative disease that leads to dementia, primarily in elderly people. The neurotoxicity of amyloid-beta (Aβ) and tau protein has been demonstrated over the last two decades. In line with these findings, several etiological hypotheses of AD have been proposed, including the amyloid cascade hypothesis, the oxidative stress hypothesis, the inflammatory hypothesis, the cholinergic hypothesis, et al. In the meantime, great efforts had been made in developing effective drugs for AD. However, the clinical efficacy of the drugs that were approved by the US Food and Drug Association (FDA) to date were determined only mild/moderate. We recently adopted a vanadium compound bis(ethylmaltolato)-oxidovanadium (IV) (BEOV), which was originally used for curing diabetes mellitus (DM), to treat AD in a mouse model. It was shown that BEOV effectively reduced the Aβ level, ameliorated the inflammation in brains of the AD mice, and improved the spatial learning and memory activities of the AD mice. These finding encouraged us to further examine the mechanisms underlying the therapeutic effects of BEOV in AD. In this review, we summarized the achievement of vanadium compounds in medical studies and investigated the prospect of BEOV in AD and DM treatment.

## 1. Introduction

Alzheimer’s disease (AD) is one of the most common neurodegenerative disorders that is estimated to currently affect 50 million people, and this number is expected to triple by 2050 [1]. Despite the intensive efforts in developing pharmaceutical agents for AD, only five have been approved by the US Food and Drug Administration (FDA) to date: cholinesterase inhibitors including Donepezil, Galantamine, and Rivastigmine, which could increase synaptic acetylcholine to improve learning and memory, and the NMDA receptor antagonist Memantine, as well as the most recently sanctified amyloid-beta (Aβ) oligomer monoclonal antibody, Aducanumab. However, the therapeutic effects of these agents are mild and quite controversial [2]. It is still an arduous task for scientists to find effective drugs for AD.

It has been revealed that diabetes and AD share some pathogenetic factors, including chronic inflammation [3,4,5], oxidative stress [5,6], adiponectin deficiency [7], abnormal expression of plasma cholinesterase [8]. Many studies demonstrated the increased risk of AD in diabetic patients and proposed a link between abnormal insulin signaling and the amyloid cascade. For example, the insulin degrading enzyme (IDE) is involved in Aβ clearance and degradation [9], and the postmortem analysis showed that IDE level was lower in brains of AD patients [10]. 

Recently, in vitro studies showed that peroxovanadium complexes could inhibit the Ab aggregation [11]. Other groups also reported that vanadyl (IV) acetylacetonate (VAC) improved the viability of neural cells under the stress of Ab [12]. In addition, we examined the therapeutic effects of vanadium compound Bis(2-ethyl-3-hydroxy-4-pyronato) oxovanadium (IV) (BEOV), which was originally synthesized as a substitution of vanadate for curing diabetes mellitus (DM) in AD models. We found that BEOV significantly reduced the levels of Aβ and tau phosphorylation, and inhibited the inflammation induced by Aβ [13], blocked the endoplasmic reticulum (ER) stress induced neurotoxicity [14], and ameliorated the spatial learning and memory in AD mouse models [15]. Moreover, we found that the biological activity of BEOV is dependent on proliferator-activated receptor gamma (PPARγ), which is similar with the activity of bis (5-hydroxy-4-oxo-4H-pyran-2-hydroxy-benzoatato) oxovanadium (IV) (BSOV) [16]. In this review, we summarize the progress of the pharmacological research on vanadium compounds, and the emerging role of vanadium compounds in treating AD.

## 2. The Pharmacological Research of Vanadium Compounds in Treating Diabetes

Vanadium is a transitional element, which exists in +2, +3, +4, and +5 oxidation states, most commonly in the tetravalent and pentavalent form. Extracellularly in body, vanadium exists in form of vanadate anion (VO3−). In contrast, intracellularly it is normally found in the form of vanadyl cation (VO2+), and associates with proteins [17].

The pharmacological research on vanadium started from an unanticipated observation that vanadate could inhibit ATPase [18]. Subsequent studies revealed the similarity of vanadate and phosphate in size and charge, which provided vanadate the abilities of irreversibly forbidding conformational change of dephosphorylate enzyme [19]. Thereafter, the insulin-like effects of vanadyl ions was perceived in rat adipocytes [20]. The peroxovanadates were estimated to be able to inhibit the insulin receptor protein tyrosine phosphatases (PTPase) [21]. The vanadyl bisacetylacetonate was shown to protect b cells from palmitate-induced cell death through inhibiting endoplasmic reticulum (ER) stress [22] and modulating PPARg activity in NIT-1 b-pancreas cells [23] and adipocytes [24]. It was also demonstrated that this vanadium compound exerted an antilipolytic effect through activating AKT [25,26]. In addition, it was found that elevated vanadium uptake could affect cholesterol and triglyceride metabolism, and stimulate hepatic glucose oxidation and glycogen synthesis [27]. However, the toxicity of vanadium was also observed in animal experiments [28,29], though the biological effects may be different dependent on the species of vanadium compounds formed in the biological media [30]. Rats would all die when they were treated with vanadyl sulfate at levels greater than 2 mM V kg^−1^ body weight [31]. Afterwards, it was determined that vanadium acts as an essential trace element in 0.05 mmol, and it will display toxicity in more the 10 mmol in animals [32]. The toxicity of vanadium is largely dependent on its oxidation state, and the highest oxidation state (+5) is the most toxic form [33]. In this condition, vanadium acted as a strong prooxidant enhancing oxidative stress [34] and perturbed mitochondria [35]. It is worth noting that the concentration of vanadium was decreased in plasma of AD patients [36,37].

In medical research, to improve the stability and affordability of inorganic vanadium salts, a series of vanadium compounds were synthesized, such as bis(maltolato) oxovanadium(IV) (BMOV) [38], BEOV [39], vanadyl complex of p-hydroxyl aminophenol derivative (VOphpada) [40], N,N-dimethylphenylenediamine-derivatized nitrilotriacetic acid vanadyl complexes (VO(dmada)) [41], and graphene quantum dots(GQD)-VO(p-dmada [42]). Oral uptake of a dose equivalent to approximately 10 mmol vanadium from BMOV had a twofold longer half-life than that from VOSO4 in bone, and 24 h after intake the absorbance of vanadium from BEOV was 2–3 times greater than that from VOSO4 in most tissues [43].

**Figure 1:**
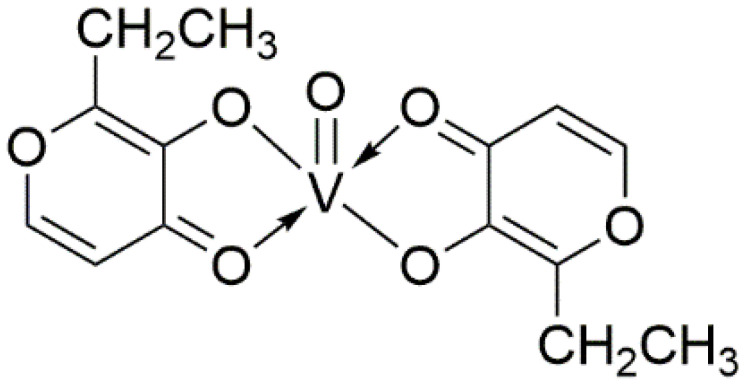
Molecular structure of Bis(2-ethyl-3-hydroxy-4-pyronato) oxovanadium (IV).

The BEOV compound contains two ethyl maltol (2-ethyl-3-hydroxy-4-pyronato) ligands and an oxovanadium, as shown by its name (Figure 1). The ethyl maltol was widely used as a food additive and flavoring agent for baked goods and some beverages [44]. Radio-labelled BEOV studies showed that most BEOV is decomposed upon entering the bloodstream [45]. In circulation, 90% vanadyl ions would be bound to citrate anions and form complexes with high molecular weight serum proteins, such as transferrin and albumin [46].

The first clinical trial using BEOV on humans was completed in 2000. Overall, no adverse health effects were seen in this trial. The safety and tolerability of BEOV doses from 10 mg to 90 mg had been confirmed [47]. A few years later, in 2007, BEOV was used in a phase IIa trial in seven type 2 diabetic patients. This study showed that oral uptake of 20 mg BEOV per day for 28 days reduced the fasting blood glucose in diabetic subjects [48]. However, in 2009 the clinical trial of BEOV was terminated. As reported, the renal changes that resulted from the preclinical study resulted in the termination of the trial [49]. Thus, the doses, the duration, and the means of vanadium compound administration need to be further examined for clinical use.

## 3. The Correlations between AD and Diabetes

It is well known that the pathological hallmarks of AD are characterized by Aβ plaques outside the neuron, which originates from the cleavage of the amyloid precursor protein (APP) by secretases and neurofibrillary tangles (NFTs) inside the neuron, which is derived from aggregated tau proteins [50]. The majority of AD cases (~95%) occurred after age 65, belonging to late-onset AD (LOAD, while the rest of (5%) AD cases which occur before 65 are considered as early onset AD (EOAD). Importantly, approximately 1–2% of AD cases are hereditary, usually carrying mutations in APP and Presenilin (PS) 1/2 (the components of secretase-gamma) genes and commonly developing AD features in the very early age of life. Most AD cases are sporadic, and aging is the most dangerous risk factor. Moreover, the initiation of AD is also bound up with some other genetic, epigenetic and environmental factors [51]. Through genome-wide association studies and other methods, it was found that many genes related to lipid metabolism, immune response and endocytosis were correlated with the occurrence of sporadic AD, including APOE, TREM2, PICALM, and CLU, et al. Among them, apolipoprotein E4 (ApoE4), which is mainly expressed by astrocyte, is the most relative genotype to AD [52]. When ApoE was knocked-out in APP transgenic animal models, they exhibited reduced fibrillar Aβ deposition and Aβ levels in their brains [53]. Thus, ApoE is likely involved in regulating the clearance of Aβ. The exact relationship between the above proteins and AD remains not fully understood, but some experiments have shown that most of the abnormal genetic factors cause Aβ overload [54]. To date, the toxicity of Aβ and tau had been well illustrated, and many hypotheses had been proposed, such as the tau hypothesis, the cholinergic hypothesis, the inflammation hypothesis, and the oxidative stress hypothesis. However, the amyloid cascade hypothesis remains the predominant etiology of AD.

An increased risk of dementia has been observed in the type 2 diabetes mellitus (T2DM) cohort [55,56]. A study involving 2500 Japanese-American diabetes subjects showed that the risk of these patients developing AD was increased by 1.8-fold [57]. In addition, the T2DM related conditions, such as obesity, hyperinsulinemia, and metabolic syndrome, were also considered as risk factors for AD [58]. Thus, the association between T2DM and AD continued to garner more attention. Though the brain used to be considered as an insulin-insensitive organ because the glucose metabolism in the brain is mainly regulated in an insulin-independent manner [59], strong evidence exists that suggests that insulin plays an important role in the central nervous system, including in cognitive behavior [60]. In addition, post-mortem studies have shown that insulin signaling was impaired in AD patients. For example, the mRNA levels of the insulin receptor were reportedly decreased in AD brains when compared to controls [61]. A reduction of insulin receptor substrate (IRS) and increased IRS-1 serine phosphorylation were noticed in brains of AD patients as well [62]. The close correlations of AD and DM may provide critical hints for searching for effective AD drugs.

### 3.1. Insulin Degrading Enzyme (IDE)

IDE is a ubiquitously expressed Zn2+ metallopeptidase. The gene of human IDE was first cloned in late 1980s [63,64]. The observations that overexpression of *Ide* in Chinese hamster ovary cells [65] and monkey kidney COS cells [66] increased the extracellular degradation of exogenously administrated insulin and reinforced the notion that insulin is a specific substrate for IDE. However, it has also been shown that insulin-like growth factor (IGF)-I and II [67], glucagon [68], Aβ [69] can also be proteolytic cleaved by IDE.

The activity of IDE is modulated by metal ions [70], ubiquitin [71], long-chain fatty acids [72] and ATP and other nucleotide triphosphates [73]. The nitrosylation on Cys812 or Cys819 can significantly inhibit the function of IDE [74]. The interaction of IDE with its substrate is also limited by the size, charge, and conformation of substrate [75]. For review, please see [76].

IDE plays an important role in insulin clearance, as evidenced by *Ide* gene ablation in mice, which resulted in impairment in the hepatic insulin clearance, followed by glucose intolerance and hyperinsulinemia [77]. Research on the polymorphisms of *Ide* revealed that the SNP rs1887922 and rs2149632 variants are associated with a higher risk of development of T2DM of about 26% and 33%, respectively [78].

Moreover, IDE is enriched in pancreatic b-cells and the brain, where the amyloidogenic risk is higher. Increased IDE nitrosylation and oxidation were found in brains of AD patients compared with age-matched healthy brains [79]. AD patients also exhibited decreased IDE level in the cortex and hippocampus [80]. It was also noticed that regions with extensive Ab deposition such as microvessels from AD patients exhibit higher levels of IDE, but with reduced activity [81]. Apparently, the overloaded Ab in AD patients can competitively bind IDE with insulin, and even result in the exhaustion of IDE. Therefore, the imbalance of insulin would further impact downstream signaling, such as the metabolism of glycogen synthase kinase 3 (GSK-3β).

### 3.2. Glycogen Synthase Kinase 3β

GSK-3 is a serine/threonine kinase that plays a central role in cell metabolism and signaling; there are two isoforms GSK-3 expressed in mammals, namely GSK-3a and GSK-3b. They are encoded by two separate genes that existed in chromosome 19 and 3, respectively, with 98% similarity. However, they apparently have different substrates and functions, as evidenced by the knockout mice; deletion of GSK-3a had no significant impact on survival, whereas a deficiency in GSK-3b resulted in severe hepatic and cardiac abnormalities causing death in embryos at E16 [82]. In addition, GSK-3b could be alternatively spliced into GSK-3b2, which contains a 13 amino acid residue insert within the kinase domains and is most abundantly expressed in the central nervous system [83].

Auto-phosphorylation on Tyr279 of GSK-3a and Tyr216 of GSK-3b resulted in constitutively active enzyme activate of these enzymes [84]. However, the activation could be arrested by other kinases. Phosphorylation on the Ser21 of GSK-3a and Ser9 of GSK3b by Akt has an inhibitory effect on GSK-3 [85,86]. Phosphorylation on the Thr390 of GSK3b by P38 mitogen-activated protein kinase (MAPK) also inactivated this enzyme [87]. On the contrary, when the phosphate is removed by protein phosphatase 2A (PP2A), GSK-3 could be reactivated [88]. GSK-3 usually exert inhibitory effects on its substrates. To facilitate the binding of GSK-3 on its substrate, a unique “priming phosphate” is needed [89]. Further research revealed that the “priming phosphate” on substrate is four amino acids behind the phosphorylate site of GSK-3 [90,91,92]. The Arg96, Arg180, and Lys205 in GSK3b form a binding pocket for the priming phosphate [93,94]. Once the phosphorylation is initiated by the “priming phosphate”, GSK-3 could carry out a multiple phosphorylation in a “relay” fashion, with the phosphorylated residue itself serving as “priming phosphate” for the next Ser/Thr within 2–5 amino acids interval [89].

Insulin resistance in T2DM is characterized by the inability in insulin response of the original insulin-sensitive tissues and reduced glucose uptake by the peripheral tissues [95]. GSK-3 is involved in glycogen synthesis and glucose uptake. It has been well demonstrated that the binding of insulin on its receptor (IR) triggers the phosphorylation of insulin receptor substrates 1 and 2 (IRS1, 2), which further activate phosphoinositol 3 kinase and in turn stimulate Akt. Therefore, insulin could inhibit GSK-3β through activating the PI3K/AKT pathway [96,97]. This further results in the decrease in phosphorylation of glycogen synthetase and its activation. However, the expression and activity of GSK-3 has been found to be increased in the skeletal muscle of T2DM patients and in the diabetes mice model [98,99], suggesting that abnormal GSK3 activity might be involved in insulin resistance and T2DM. 

Interestingly, the level of GSK-3b, which is most abundantly expressed in the brain, increases with age [100]. Moreover, the dysregulation of GSK-3b was also observed in AD patients [101]. Previous studies have illustrated that presenilin 1, one of the catalytic components of g-secretase complex, is a substrate of GSK-3b [102]. In addition, it was found that the level of beta-site amyloid precursor protein cleaving enzyme-1 (BACE-1) was also upregulated by GSK-3b [103]. Thus, it is not unexpected that the level of Ab is increased along with the elevation of GSK-3b activity [104]. More importantly, the phosphorylation of tau was strongly impacted by GSK-3β [105]. Tau is a microtubule-associated protein; the affinity of tau to microtubules depends on its phosphorylation status. In AD, the hyper-phosphorylation of tau affected the interaction between tau and the microtubule, and facilitated the accumulation of tau-filament [106].

The protective effects of GSK-3b inhibitors have been tested in the AD model, and many studies showed positive results in AD animal models [107,108,109] (for review please see [110]). However, since GSK-3β is widely expressed and implicated in many singling pathways, the side effects of its inhibitor may raise concerns for long-term treatment. 

### 3.3. Ferroptosis

Ferroptosis is a novel identified form of programed cell death which was first described in 2012 [111]. In brief, ferroptosis is characterized by excessive lipid peroxides that are induced by either Fenton reaction of polyunsaturated fatty acids (PUFA) or the iron-catalyzed enzymatic reaction of lipid oxidation. The lipid peroxides further result in the mitochondrial shrinkage, mitochondrial bilateral membrane thickening and rupture, and intracellular NADPH depletion [112]. Glutathione peroxidase 4(Gpx4) is responsible for lipid peroxide scavenging in cells, thus exerting an inhibitory effect on ferroptosis. Given that the reduction of Gpx4 is dependent on glutathione, which consists of glutamate, glycine, and cysteine. The disfunction of *x*-c system that involved in cellular cysteine supply can accelerate ferroptosis [112].

It has been confirmed that the circulating iron and ferritin levels are significantly elevated in patients with T2DM [113,114]. Studies also revealed that patients with T2DM are devoid of GSH, especially if microvascular complications are present [115]. In addition, high glucose could reduce SOD and Gpx4 activity [116] and result in iron overload in b-cells, thus giving rise to ferroptosis [117].

In the late-stages of AD, the enlargement of cerebral ventricles resulting from neuron death is one of the most desperate events in AD pathology. In the past, it was mainly attributed to the toxicity of Ab [118], tau propagation [119] and inflammation [120]. However, recent studies demonstrated that iron was accumulated in brain of AD patients [121,122]. The level of mitochondrial ferritin was significantly increased in the frontal cerebral cortex in AD patients. Besides the increased iron, accumulated lipid ROS and decreased mitochondrial ferritin and cortical GSH content have also been found in the AD pathology [123].

The mechanisms of how ferroptosis occurred in AD is unclear so far, whether it is corelated with the dyshomeostasis of glucose metabolism is an interesting question to contemplate. However, the inhibitor of ferroptosis has already shown benefits in pre-clinical trials. For example, the ferrotortin-1 liproxstatins-1 were found to be effective in reducing the neuronal death and memory impairment induced by Ab aggregation in vitro and in vivo. Moreover, the supplication of selenocystein, which is the active center of Gpx4 also ameliorated the AD pathology in mice models [124,125].

## 4. The Protective Efficacy of Vanadium Compound on AD Mouse Models

In previous studies, the APPswe/PS1De9 (strain name: B6C3-Tg(APPswe, PSEN1dE9)) AD model mice were first adopted to evaluate the effects of BEOV [15] and VAC [12]. This mouse strain carried familial AD mutations of APP and PS1, which are APP KM670671NL (Swedish mutation) and PS1 deltaE9. They begin to develop Aβ deposits by six months of age, with abundant plaques in the hippocampus and cortex by nine months. Plaques continue to increase up to 12 months of age [126]. The neuronal loss could be seen at eight to ten months [127]. The spatial learning activity in the Morris water maze is comparable to non-transgenic mice at seven months of age, but impaired by 12 months [128]. BEOV were given at 0.2 mmol/L or 1 mmol/L in daily drinking water for three months from six to nine months of age. The daily BEOV uptake was estimated ~0.2 mg or ~1 mg, respectively. By using 18F-labled fluorodeoxyglucose positron emission tomography (18F-FDG PET) to determine the glucose metabolism, it was found that BEOV treatment increased the glucose uptake in the APPSwe/PS1De9 mice vs. control. It was also shown that BEOV significantly reduced Aβ levels in the hippocampus and cortex. In addition, BEOV also promoted the clearance of Aβ through autophagy, as evidenced by the decreased light chain protein 3-II (LC3-II)/LC3-I ratio [15].

Further studies revealed that BEOV attenuated the neuroinflammation evoked by Aβ. The administration of BEOV significantly reduced the levels of inflammatory factors, including tumor necrosis factor-a (TNFa), interleukin-6 (IL6), and interleukin-1b (IL1b), both in AD mice brains and in Aβ threatened BV2 microglia. The activation of nuclear factor-kB (NF-kB) was suppressed by BEOV via proliferator-activated receptor gamma (PPARg). This was demonstrated by GW9662, a PPARg inhibitor, which eliminated the above effects of BEOV in vitro [13]. These effects of BEOV are similar with insulin in regulating insulin receptor substrates (IRS), which subsequently activate AKT. In addition, it was also shown that BOEV attenuated the neurotoxicity of Aβ by suppressing the levels of immunoglobulin protein GRP78 (also known as bip), and C/EBP homologous protein-10 (CHOP), a pro-apoptotic signaling factor triggering programmed cell death [14].

Moreover, the triple transgenic AD (3xTg AD) model mice (name: B6; 129Psen1*tm1Mpm* Tg(APPSwe, TauP301L)), which carried the human APP KM670671NL, tau P301L, and PS1 M146V mutation, were also recrewed to examine whether the tau pathology was affected by BEOV. These mice are from the widely-used homozygous familial AD model. They display Aβ plaques by six months of age in the frontal cortex, and these become more extensive by 12 months of age. The NFT occurred by 12 to 15 months [129]. At age 6.5 months, these mice exhibited learning and memory defects in a Barnes maze [130]. It was shown that BEOV significantly improved the spatial learning behavior of 3xTg AD mice in a Morris water maze. In addition, both 0.2 mg and 1 mg BEOV reduced the Aβ level in mice model brain. This effect is mediated by both PPARg and protein tyrosine phosphatase 1B (PTP1B). More importantly, the phosphorylation of tau protein, which is generally related the dissociation of tau from tubulin and a prelude of NFT formation, was decreased by BEOV treatment. This was due to the inhibition of GSK3β by PTP1B inactivation [131]. These results are coincident with the observations that GSK3β was inhibited by jamunone M through PTB1B [132]. The evidences of the therapeutic effects of BEOV on different AD mice models is summarized in Table 1.

## 5. Conclusions and Perspectives

In the past few years, the similarity of the impaired glucose metabolism in AD and DM has drawn more and more attention. AD was even characterized as type 3 diabetes [133]. The mechanisms underlying the disfunction of glucose metabolism in both diseases may provide a new therapeutic approach for AD. The clinical studies showed that the vanadium compound truly improved the glucose uptake in DM patients; on the other hand, the experimental evidence showed that vanadium compounds could also regulate the level of Ab in vivo and in vitro [12,15].

**Figure 2:**
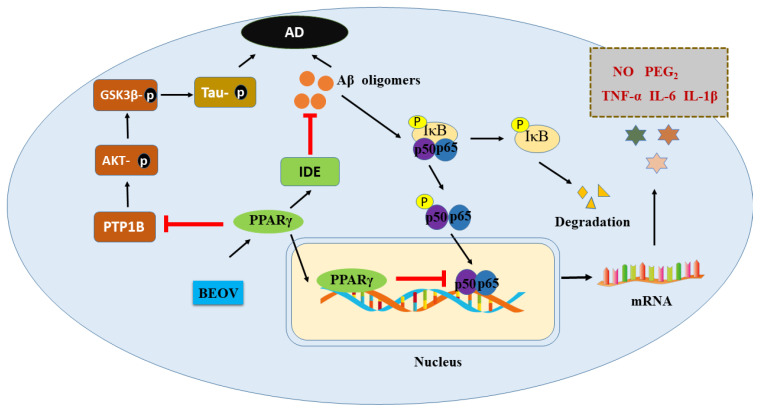
Schematic illustration of the potential mechanism underlying preventive role of BEOV in AD pathology.

As mentioned above, the protective effects of vanadium compound in AD models were found to be mediated by PPARg, though which BEOV antagonized the activity of NF-kB, which is characterized by the decrease of TNF-a and IL-6, thus inhibiting the inflammation induced by Ab [12,13]. Moreover, BEOV also reduced the phosphorylation of tau in a triple transgenic AD mice model [131] via its inhibitory effects on PTP1B and GSK-3b afterward [131]. Many studies have demonstrated that PPARg exert an important role in glucose homeostasis and insulin sensitization [134,135]. Interestingly, it has been reported that the level of IDE, which is involved in the clearance of insulin and Ab, is also upregulated by PPARg [136]. Therefore, vanadium compound may elevate the level of IDE in the AD model through activating PPARg, in turn facilitating the clearance of Ab (Figure 2).

The results derived from the mouse model robustly proved that the vanadium compounds were beneficial for the central nervous system [137], and could be considered as potential drugs for curing AD [11,12,138]. In addition, another vanadium compound, bis-[curcumino]oxovanadium (BCOV) [139], which was synthesized by curcumino and oxovanadium, also displayed protective effects on diabetes mellitus [140]. It was shown that BCOV could reduce the serum LDL level significantly in obese rats [140]. Curcumin is both an antioxidant and an antidiabetic agent [141]; it had been reported that curcumin inhibited the oligomerize of tau [142]. This vanadium compound is deserved to be investigated in further studies in order to determine the therapeutic effects of vanadium compounds on AD. It has been reported that vanadium in the form of vanadyl (VO2+) binds to transferrin at the same binding site as the Fe3+ ion [143]. Whether a vanadium compound could exert any effects on ferroptosis in neurodegenerative disease is an attractive subject as well.

## Figures and Tables

**Table 1:** The effects of vanadium compound in different AD models.

Name AD Models	AD Pathology in Models	The Effects of Vanadium Compounds
N2A cell line Swedish mutation of APP	Increased Ab burden	BEOV decreased Ab level [13,15], increased the expression of PPARg
SY5Y cell line with Swedish mutation of APP	Increased Ab burden	VAC elevated the levels of PPARg, AMPKa and GSK-3b [12]
Tg(APPswe, PSEN1dE9)	Ab senile plaques, spatial learning impaired begin on 12 months of age.	BEOV [13,15] and VAC [12] improved spatial learning activity in Morris water maze, decreased Ab level, increased neuron viability.
Tg(APPSwe, TauP301L)	Ab senile plaques and tau filaments tangles, spatial learning activity impaired begin on 6.5 months of age.	BEOV improved spatial learning activity in Morris water maze, decreased Ab level and tau phosphorylation, increased neuron viability [131]
